# 23. FRONTAL CORTEX DEVELOPMENT AND RISK FOR PSYCHOPATHOLOGY: MOLECULAR AND GENETIC MEDIATORS AS POSSIBLE BIOMARKERS?

**DOI:** 10.1093/schbul/sby014.090

**Published:** 2018-04-01

**Authors:** Francesco Papaleo

**Affiliations:** Instituto Italiano Di Tecnologia

## Abstract

**Overall Abstract:** This panel includes 4 females and 1 male, 2 early career scientists, 2 clinicians. From 5 different countries, 3 different continents.

Prefrontal cortex (PFC) dysfunction is associated with alterations in cognitive processing impaired in schizophrenia. The development of the PFC is a protracted process, which peaks in adolescence and ends only in early adulthood. Its extended development renders the PFC particularly susceptible to environmental influences, but we know very little about the underlying neurobiological mechanisms. More importantly, we need to understand how risk or protective factors can affect PFC development. This could have an impact towards the development of early and/or preventive treatments for cognitive dysfunctions relevant to a number of psychiatric disorders including schizophrenia.

We will discuss recently-discovered processes involved in different stages of prefrontal cortex development, including gestation and adolescence, and how alterations to these events may lead to schizophrenia-relevant phenotypes. A multidisciplinary group of preclinical and human researchers will discuss recently-identified molecular, genetic, and hormonal events that shape PFC development. We will also show compelling new evidence that disruption to these developmental processes is linked to psychiatric conditions. The data we will present include:

(1) Signaling events implicated in the migration of newborn neurons into emerging cortical layers and their relevance to schizophrenia and autism spectrum disorder (Helen Cooper).

(2) Mechanisms related to adolescent axonal growth and connectivity in the PFC and how they are disrupted by ongoing experiences (Cecilia Flores).

(3) Findings from human and mouse studies regarding the impact of the 22q11.2 microdeletion on PFC development and cognitive maturation (Francesco Papaleo).

(4) The molecular development of the postnatal human cortex, including the maturation of interneurons, molecular changes in neurotransmitter signaling pathways, synaptic development and developmental changes in those immune related molecules that may impact synaptic development (Maree Webster).

Collective discussion of these data (Maude Schneider) will highlight important implications for prevention and early intervention strategies in schizophrenia.

